# Transitional feeding in developing pigs: Effects of food texture modification on liquid swallowing behavior and epiglottic movements

**DOI:** 10.14814/phy2.70878

**Published:** 2026-04-17

**Authors:** Stephane J. Montuelle, Donna R. Scarborough, Hannah R. Baker, Susan H. Williams

**Affiliations:** ^1^ Department of Biomedical Sciences Ohio University Heritage College of Osteopathic Medicine Warrensville Heights Ohio USA; ^2^ Department of Speech Pathology and Audiology Miami University Oxford Ohio USA; ^3^ Cincinnati Children's Hospital Medical Center Liberty Ohio USA; ^4^ Department of Biomedical Sciences Ohio University Heritage College of Osteopathic Medicine Athens Ohio USA

**Keywords:** airway protection, gape cycle, international dysphagia diet standardization initiative, pediatrics, weaning

## Abstract

Food texture modifications facilitate the transition from suckling to chewing. We examined drinking and liquid swallowing in 2 groups of pigs: one group directly transitioned from liquids to solids (DT) and the other transitioned progressively (PT) from liquids to soft foods then to solids. Our study reports the typical outcome of maturation of oropharyngeal physiology in both groups with similarities in the behavioral aspects of liquid swallowing (swallows occur during ingestion cycles, similar cycle frequency). However, compared to DT pigs, PT pigs used fewer gape cycles and less time between swallows and initiated the swallow later in the gape cycle, which are characteristics of swallow in younger pigs. Swallows in PT pigs were also typically faster with shorter epiglottal descent duration to reach laryngeal vestibule closure (LVC) quicker, thus increasing LVC duration significantly relative to swallow duration. Furthermore, throughout the study, epiglottal descent duration decreased in PT pigs but increased in DT pigs, emphasizing how each transition strategy altered the maturation of a key component of the swallow. Our results expand our understanding of liquid swallowing behavior in mammals and showcase how food‐texture modification during a critical period of development can impact the maturation of swallow physiology.

## 1 INTRODUCTION

From birth, all mammals ingest a liquid diet which contains essential nutrients, immunoglobulins, and hormones for adequate growth and development (Power & Schulkin, 2013). However, as an infant's nutritional needs overtake what can be provided solely through liquid intake, the infant must be introduced to complementary foods of increasing texture and nutritional value. Furthermore, in humans, critical periods for developing feeding skills necessary to consume a diverse diet occur in the second half of the first year of life, coinciding with rapid anatomical, neuromotor, and sensorimotor maturation (Robbins et al., 2008; Drzewicki et al., 2025). Failure to acquire these skills through this transitional feeding time frame, which stimulates oral motor and sensory systems, negatively impacts oromotor function and control (Coulthard et al., 2009; da Costa et al., 2017; Northstone et al., 2001; Sdravou et al., 2023). Recently, a systematic approach for examining the various textures of foods and liquids was developed that allows for a point‐of‐feeding textural determination that can be applied to this transitional period. The International Dysphagia Diet Standardization Initiative (IDDSI) framework introduced both standardized descriptions of food textures and readily accessible, low‐tech clinical testing measures that allow for the classification of food textures into objective categories (Cichero et al., 2013; Cichero et al., 2017). Leveraging the application of the IDDSI framework in controlled studies using validated animal models for swallowing provides an opportunity to examine potential developmental physiological changes with systematic exposure to different consistencies. This framework is essential for understanding developmental norms related to swallowing in humans during the critical periods of transitional feeding.

The pharyngeal phase of the swallow uses a series of highly coordinated events to ensure passage of the bolus into the esophagus while safely protecting the airway. In the adult human, Martin‐Harris and colleagues (Martin‐Harris et al., 2008) identified seventeen unique physiological components for successful swallowing. One of these components, epiglottal movement, has presented a conundrum over the past several decades regarding the mechanism of action particularly related to anatomic position and movement. The epiglottis of the adult human is spatially positioned relatively inferiorly in the neck compared to other mammals, with a clear separation during swallowing from the soft palate to allow for epiglottal folding or inversion to cover the airway. However, in human infants and most mammals the epiglottis and soft palate are in close proximity or touching. In some mammals, the epiglottis rests on the soft palate and the larynx is in an intranarial position, opening directly into the nasopharynx (Laitman & Reidenberg, 1993). Because of the anatomic coupling of these two structures, this configuration was hypothesized to allow for swallowing and respiration simultaneously without inversion of the epiglottis (Laitman et al., 1977). A landmark paper in two diverse adult mammal populations by Larson and Herring (Larson & Herring, 1996) revealed that despite the superior spatial positioning of the epiglottis, respiration ceases during swallowing and that the epiglottis inverts completely during every swallow regardless of the consistency of the bolus. Interestingly, since this early work, Crompton, German, and Thexton (Crompton et al., 2008) have documented a developmental continuum of epiglottal movement patterns from infancy to adulthood in pigs. In infant pigs, the liquid bolus can traverse lateral to the larynx while the epiglottis remains in an intranarial position. As the piglets are being weaned, a “transitional” swallow is described where the epiglottis inverts relatively slowly and the bolus never moves over the tip of the epiglottis. Finally, for post‐weaning juvenile and adult pigs both liquid and solid boluses traverse over a rapidly inverted epiglottis.

This paper focuses on drinking and liquid swallowing behaviors of weaned piglets to understand whether, and if so, how feeding practices after prolonged exposure to liquidized diet influence the development and maturation of behavioral and physiologic parameters related to drinking and liquid swallowing, particularly related to airway protection. We examine two groups of healthy weaned piglets that were first maintained on a liquidized diet, and then transitioned to solid foods in two different ways: (1) direct transition to solid foods or (2) gradual transition from liquid to soft solids to solid foods. Although the primary goal of modifying food texture is to facilitate the transition to swallowing solid foods safely, this study focuses on drinking behavior, and feeding behavior proper will be examined separately. Liquid drinking was selected because, compared to solid foods, liquids require minimal intraoral processing and are arguably ready to be swallowed as soon as they are ingested. As such, investigating the swallowing of liquids directly assesses the effects of the two different transition strategies on swallowing, without the potential artifacts that the two transition strategies could have on the manipulation and processing of solid food boluses. Furthermore, because food texture modification is primarily implemented to facilitate the acquisition of the motor skills necessary to swallow solid foods safely, we expect both transition strategies (i.e., direct vs. progressive) to have minimal effects on the behavior and movements during drinking. Confirming these expectations would indicate that both food transition strategies do not adversely affect the development and maturation of liquid swallowing.

## 2 MATERIALS AND METHODS

### 2.1 Experimental structure and timeline

Eleven pre‐weaning Berkshire piglets (Sus scrofa Linnaeus 1758; 10 females, 1 male) were obtained at the age of 3 weeks. From 3 until 12 weeks of age, all individuals were fed pulverized Labdiet Grower pellets (Purina Labdiet, Gray Summit, MO, USA) mixed with water at a ratio of 100 g of pellet to 240 mL water. This produces a homogeneous “liquidized” texture reflecting level 3 of the International Dysphagia Diet Standardization Initiative (IDDSI) (Cichero et al., 2017; IDDSI, 2019). IDDSI levels are mainly defined qualitatively, with level 3 referring to “moderately thick” that (i) drips slowly in dollops through the prongs of a fork, or (ii) no less than 8 mL should remain in a 10 mL syringe after 10 s of flow. After baseline data collection at 12 weeks, individuals were randomly assigned to 2 groups: direct transition (DT) or progressive transition (PT). The direct transition group was then fed only solid pellet chow until data collection at 16 weeks of age, whereas the progressive transition group was gradually transitioned to solid foods over the course of 4 weeks (i.e., until 16 weeks of age) using weekly incremental increases in food texture following IDDSI levels, culminating with solid pellet chow equivalent to IDDSI level 7. Water was always available ad libitum. All procedures and experiments involving live animals were monitored and approved by the Ohio University Institutional Animal Care and Use Committee (protocol #22‐U‐001).

Between 3 and 16 weeks of age, pigs undergo a critical developmental period marked by changes in orofacial morphology (e.g., bunodont tooth eruption) and maturation of key oropharyngeal functions (e.g., weaning, chewing dynamics, tongue lateralization) (Herring, 1985; Thexton et al., 1998). This critical period is paralleled in humans during the first few years of life (Delaney & Arvedson, 2008; Green et al., 1997). At the end of our study, the pigs are comparable to a 2–2.5 year old child who should be independently eating a variety of foods with different textures. Therefore, maintaining all pigs on a liquidized diet through 12 weeks simulates a significantly protracted food texture modification regimen that clinically reflects facilitation of liquid to solid food transition in a population of infants and children with feeding difficulties during this critical period of oral development. In our study, the direct transition strategy not only minimizes the delay in exposure to solid foods, it mirrors weaning practices of direct exposure to a regular diet, whereas the progressive strategy parallels more traditional complementary feeding practices (Cichero, 2016). This design also means that, at 16 weeks of age, the direct transition group had 4 weeks of experience manipulating and processing solid foods, whereas 16 weeks in the progressive transition group corresponds to only 1 week of experience with solid foods.

### 2.2 Data collection

Data were collected at 12 and 16 weeks of age in all 11 individuals, with data collected at 12 weeks reflecting baseline data before the transition strategies were implemented. All data collected at 16 weeks correspond to the end of each transition strategy. Prior to data collection, a tantalum ligating clip (Teleflex® Hemoclip® #523335, Wayne, PA, USA) was attached to the epiglottis of each animal under light isoflurane anesthesia. High‐speed video sequences were recorded with two synchronized Miqus cameras (Qualisys, Goteborg, Sweden) mounted on the output ports of the image intensifiers of two synchronized OEC‐9000 fluoroscopes (General Electric, Boston, MA, USA; refurbished by Imaging Systems and Services Inc., Painesville, OH, USA). Sequences were recorded at 200 frames/s for a total duration of 60 s, with radiation doses averaging 80 kVp and 4.5 mA across trials. All sequences for each animal were recorded in a single day to minimize experience and limit the potential for motor learning. For each sequence, 400 mL of water was mixed with 52 mL of milk replacer and powdered barium (EZ Paque Barium Sulfate Powder, Bracco Diagnostics Inc., Milan, Italy) to enhance contrast on the x‐ray images and allow for visualizing the position of the bolus within the oral cavity.

### 2.3 Variables

Video sequences were analyzed visually to extract frame numbers of key events, which were then converted into timings and durations. The frame of first contact between the tongue and the food was used as the initial start time of the drinking sequence. All timing variables were determined relative to the initial start time so that they were comparable between sequences within and across individuals. Drinking sequences consist of a series of cyclical small‐amplitude jaw opening‐closing cycles, or gape cycles, that occur in coordination with small amounts of tongue protrusion and retraction. For most cycles, gape never fully closes, and the tongue tip remains outside the oral cavity proper (i.e., the space bordered anteriorly by the teeth) (Olson et al., 2021; Thexton et al., 1998). Each gape cycle was identified as the period between successive instances of maximum jaw opening. Gape cycle duration was converted from frame numbers into seconds. Two variables were extracted at the level of the drinking sequence: cycle frequency (number of gape cycles/second) and average swallow frequency (number of swallows/second). Additionally, all swallows were evaluated using the 7‐point Infant Mammalian Penetration‐Aspiration Scale (IMPAS) (Holman et al., 2013).

Each gape cycle was classified as an ingestive or transport cycle. Cycles in which the tongue tip contacted the liquid were categorized as ingestion. Cycles in which the tongue tip did not contact the liquid but the cyclical movement of the jaw continued were categorized as transport. In this classification, ingestion cycles include some of the liquid bolus being transported through the oral cavity, whereas transport cycles do not include any ingestion of new liquid. Then, swallows, which always occurred during ingestion or transport, were identified as the cycles in which the epiglottis inverted and returned to its resting position on the soft palate and the passage of the bolus into the esophagus was clear. This distinction allowed us to quantify the proportion of swallows that occur during one or the other behavior and may be important for understanding variation in cycle duration. Three variables were quantified for each swallow: the number of gape cycles preceding the swallow, the latency between 2 consecutive swallows, and the duration of the gape cycle during which the swallow occurred.

Bolus volume was also approximated for each swallow as follows. First, the total volume ingested during a sequence was calculated as the difference between the initial volume of liquid at the start of each sequence, and the volume of liquid remaining at the end of each sequence. Then, the total volume ingested during a sequence was divided by the number of ingestion cycles to provide the average volume ingested during each ingestion cycle. Finally, the average volume ingested per cycle was multiplied by the number of ingestion cycles preceding each swallow to approximate the bolus volume associated with each swallow.

**FIGURE 1:**
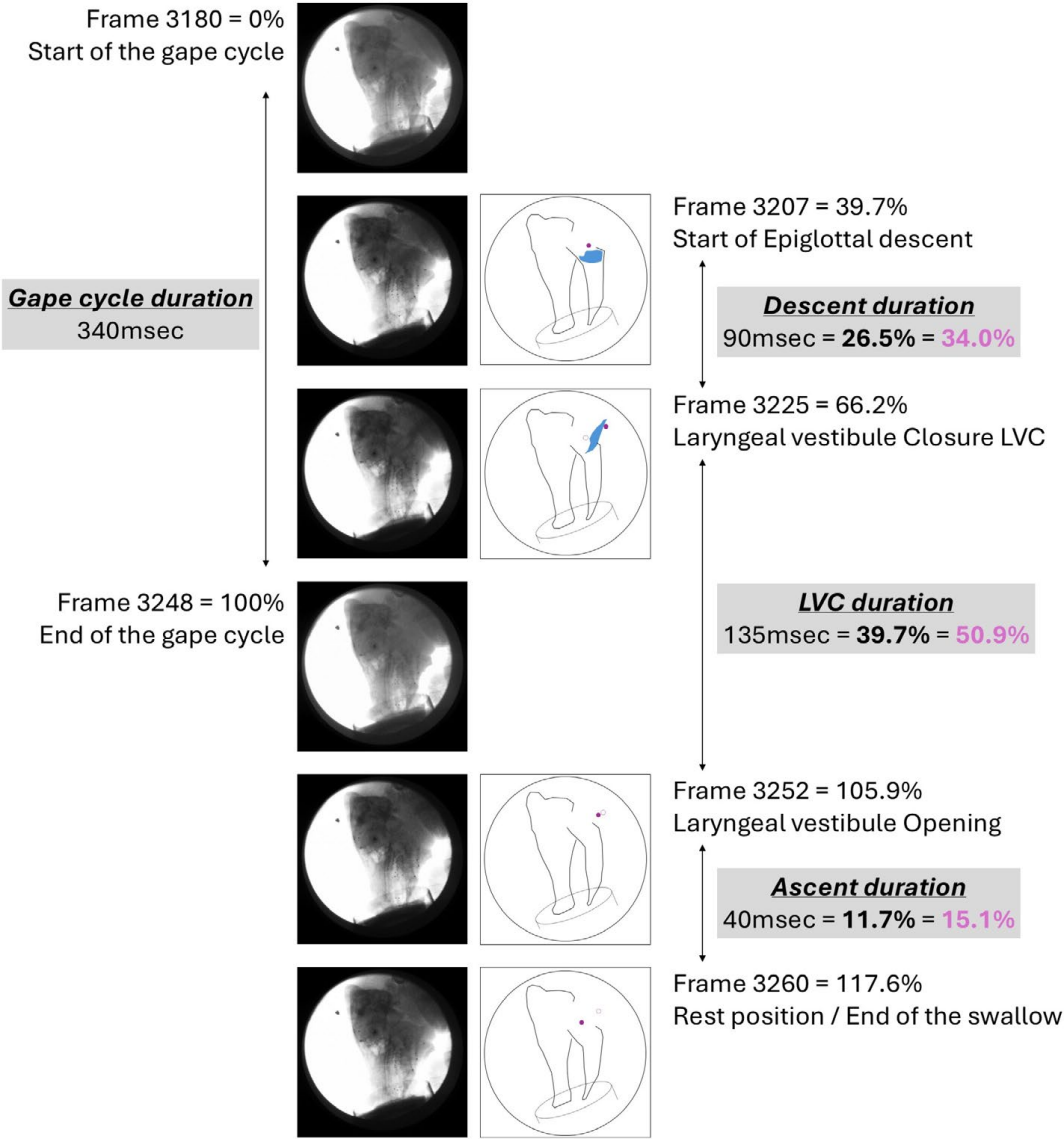
Representative frames demonstrating the timepoints extracted for analysis. From the top: Time 0: Start of the gape cycle; T1: Start of epiglottal descent; T2: Laryngeal vestibule closure (LVC); T3: End of the gape cycle; T4: Laryngeal vestibule opening; and T5: Epiglottis in rest position. Schematics on the right highlight the position of structures of interest at key frames from the corresponding fluoroscopy images on the left as follows: Black, skull and jaw; dashed line, bowl; blue, liquid bolus; pink closed circle, position of the epiglottis marker; pink open circle, position of the epiglottis marker at the previous frame to illustrate the corresponding frame‐to‐frame movement. All data were expressed as % of gape cycle duration (bold) and as % of total epiglottal movement (pink).

Within each swallow, the timepoints of 4 key epiglottal positions were extracted (Figure 1) as follows: (1) onset of epiglottal descent: first frame when the epiglottis disengages from the soft palate; (2) fully inverted epiglottal position corresponding to start of laryngeal vestibule closure (LVC): the first frame where the epiglottis reaches its caudal‐most position (e.g., Gross et al., 2018); (3) onset of epiglottal ascent corresponding to laryngeal vestibule opening (LVO) thus the end of LVC; and (4) epiglottal reset: the first frame where the epiglottis is back to its rest position on the soft palate. Based on these 4 timepoints, 4 durations were calculated: (a) total duration as the difference between the onset and end of epiglottal movement (between timepoints 1 and 4), (b) descent duration as the difference between onset and LVC (between timepoints 1 and 2), (c) LVC duration from when the epiglottis reaches its caudal‐most position to protect the airway, until the laryngeal vestibule reopens (between timepoints 2 and 3), and (d) ascent duration as the time difference between when the laryngeal vestibule opens and the epiglottis comes back to its rest position (between timepoints 3 and 4). All timing and duration variables were then converted in % of gape cycle duration to account for variability in cycle duration between swallows within and across all individuals (Figure 1). Converting epiglottal parameters relative to the gape cycle evaluates the time coordination of epiglottal movements with respect to jaw movements, thus exploring the ecological validity of these parameters for intercalated swallows, which may not be the case for isolated swallows. Durations were also standardized in % of total duration of epiglottal movement to explore the time allocated to each of the three stages: descent, LVC, ascent/reset to rest.

### 2.4 Statistical analyses

Differences between the DT and PT groups were tested for each variable at 12 weeks to determine whether both groups were comparable at the start of the transition experiment. This finding was confirmed with an analysis of variance with mixed‐effect models coupled with F‐ratios (lmer function in the lme4 R‐package; version 1.1–37). The Treatment factor was entered as the fixed factor and the Individual factor was the random factor. Second, differences between the groups at 16 weeks were tested for each variable with an analysis of variance (aov function in stats R‐package; version 4.5.2) coupled with F‐ratios. In that analysis, the Treatment factor was entered as the fixed factor. Given that each individual only belongs to one treatment group, the individual factor was nested in each treatment group. Additionally, in all analyses (i.e., at 12‐ and 16‐weeks), effect size was computed using the eta_squared function in the effectsize R‐package (version 1.0.1), and bolus volume was included as a covariate. All analyses were performed in R (version 4‐4‐3). All the sequences recorded for each individual were included in the final analysis, and the final data sample is presented in Table 1. To minimize analytical bias, pig identification numbers were randomly assigned within each experimental group to eliminate any systematic association between individual identifiers and treatment allocation. Additionally, the name of the video sequences themselves only included the individual ID, with no reference to treatment group, thereby ensuring that evaluators remained blinded to treatment conditions during analysis.

**TABLE 1:** Summary of the data sample used in this study. Treatment groups (PT, progressive transition, DT, direct transition), sex (M, male, F, female), and weight at 16 weeks are all specified for each individual.

Animal ID (sex, weight@16 weeks)	12 weeks	Treatment group 13–16 weeks	16 weeks
Pig 40 (M; 33.2lbs)	1 sequence/26 swallows	PT	1 sequence/27 swallows
Pig 43 (F; 25.0lbs)	1 sequence/36 swallows	DT	1 sequence/21 swallows
Pig 45 (F; 17.8lbs)	1 sequence/45 swallows	PT	1 sequence/43 swallows
Pig 48 (F; 14.6lbs)	2 sequences/43 swallows	DT	3 sequences/29 swallows
Pig 49 (F; 12.2lbs)	1 sequence/37 swallows	PT	1 sequence/28 swallows
Pig 52 (F; 34.6lbs)	1 sequence/37 swallows	PT	2 sequences/29 swallows
Pig 53 (F; 39.0lbs)	3 sequences/61 swallows	DT	1 sequence/25 swallows
Pig 54 (F; 39.4lbs)	3 sequences/49 swallows	DT	1 sequence/26 swallows
Pig 55 (F; 39.0lbs)	5 sequences/75 swallows	DT	1 sequence/18 swallows
Pig 56 (F; 48.0lbs)	5 sequences/42 swallows	PT	1 sequence/26 swallows
Pig 57 (F; 43.0lbs)	2 sequences/23 swallows	PT	1 sequence/21 swallows
Total sample	25 sequences/474 swallow	DT PT	7 sequences/119 swallows 7 sequences/174 swallows

## 3 RESULTS

Even though the effects of direct versus progressive transition (DT and PT) were assessed with the data collected at the completion of the experiment (i.e., 16 week data), this section also presents the 12 week data as a baseline to showcase the trend in which variables changed over the course of the 4‐week experiment. Group averages are presented in Tables 2 and 3, and individual averages are available in Tables S1–S6. All individuals included in this study were healthy throughout the experiment and demonstrated normal growth and maturation. In addition, based on IMPAS scores, we did not observe aspiration or penetration in any of the swallows recorded at 12 and 16 weeks, indicating that swallow safety was unaffected. Prior to being randomized into the treatment groups at 12 weeks, the two groups were not significantly different for any of the variables (see Tables S1–S6). Therefore, between‐group differences observed at 16 weeks can be confidently interpreted as the effect of the two treatment strategies. In addition, bolus volume was not significantly different between the two transition strategies (Table 2), but was a significant covariate for a number of duration and timing variables (see details below).

**TABLE 2:** Summary of the behavioral variables during drinking and swallowing in 12‐ and 16‐week old pigs. Table entries are group averages (± standard deviation), with associated statistical results. Detailed individual averages and between‐individuals differences are provided in Tables S1–S3.

	12 weeks	16 weeks		16 week DT vs PT
	All individuals	DT	PT	*F*_(df1,df2)_, *p*
Drinking sequence variable				
Cycle frequency (cycles per sec)	3.20 ± 0.26	2.71 ± 0.23	2.89 ± 0.38	*p* = 0.296
Swallow variables				
Swallow frequency (swallows per sec)	0.76 ± 0.35	0.35 ± 0.20	0.53 ± 0.25	*p* = 0.155
Bolus volume (mL)	8.6 ± 9.4	10.8 ± 11.1	9.2 ± 11.0	*p* = 0.196
Latency between 2 swallows (sec)	1.09 ± 1.58	2.54 ± 3.07	1.28 ± 1.52	***F***_**1,276**_ **= 39.45** ***p* < 0.0001**
Number of cycles before a swallow	3.2 ± 4.9	6.7 ± 7.6	3.2 ± 3.9	***F***_**1,276**_ **= 52.84** ***p* < 0.0001**
Swallow cycle variables				
% ingestion swallows	95.4%	88.2%	100%	‐
% transport swallows	4.6%	11.8%	0%	‐
Ingestion Swallow Cycle Duration	329 msec ±50	365 msec ±94	364 msec ±66	*p* = 0.907
Transport Swallow Cycle Duration	398 msec ±79	485 msec ±41	NA	‐
Non‐swallow cycle variables				
Ingestion/Transport Cycle Ratio	89.0% ingestion 10.0% transport	82.6% ingestion 17.4% transport	96.8% ingestion 3.2% transport	‐
Ingestion Cycle Duration	298 msec ±56	361 msec ±100	350 msec ±102	*p* = 0.058
Transport Cycle Duration	364 msec ±70	417 msec ±89	427 msec ±99	*p* = 0.613

*Note*: Bold values highlight the variables where there was a significant difference between the 2 groups.

**TABLE 3:** Summary results of the epiglottis inversion parameters during liquid swallowing in 12‐ and 16‐week old pigs. All timings and durations were converted as a % of gape cycle duration. Table entries are group averages (± standard deviation), with associated statistical results. Detailed individual averages and between‐individuals differences are provided in Tables S4 and S5.

	12 weeks	16 weeks		16 week DT vs. PT
	All individuals	DT	PT	*F*_(df1,df2)_, *p*
Relative durations (% cycle duration)				
Total	74.8% ± 12.6	77.6% ± 22.9	69.4% ± 12.9	***F***_**1,290**_ **= 15.43** ***p* = 0.0001**
Descent	21.1% ± 8.3	23.6% ± 9.0	18.3% ± 7.1	***F***_**1,290**_ **= 31.70 *p* < 0.001**
LVC	42.1% ± 9.2	45.5% ± 17.3	41.7% ± 8.0	***F***_**1,290**_ **= 6.43** ***p* = 0.0001**
Ascent	11.6% ± 5.0	8.6% ± 4.9	9.5% ± 2.9	*p* = 0.059
Relative timings (% cycle duration)				
Time to the start of epiglottal descent	49.0% ± 11.3	38.7% ± 15.8	51.6% ± 13.1	***F***_**1,290**_ **= 58.45 *P* < 0.0001**
Time to LVC/end of epiglottal descent	70.2% ± 13.3	62.2% ± 15.6	69.8% ± 13.1	***F***_**1,290**_ **= 20.69 *p* < 0.0001**
Time to LVO/start of epiglottal ascent	112.0% ± 13.9	107.0% ± 14.4	112.0% ± 6.5	***F***_**1,289**_ **= 6.33** ***p* = 0.012**
Time to the end of epiglottal ascent	124.0% ± 16.1	116.0% ± 19.6	121.0% ± 17.2	***F***_**1,290**_ **= 4.69** ***p* = 0.031**

*Note*: Bold values highlight the variables where there was a significant difference between the 2 groups.

### 3.1 Sequence‐level data

At the start of the experiment at 12 weeks, pigs drank at a frequency of approximately 3 gape cycles per second, with swallowing occurring at an average rate of 0.76 swallows per second (Table 2). Between 12 and 16 weeks, drinking cycle frequency decreased in both DT and PT pigs, which reflects an increase in cycle duration as they physically matured (see below). Similarly, swallow frequency also decreased between 12 and 16 weeks in both DT and PT groups, indicating more time between swallows in 16‐week old pigs, which is likely associated with an increase in the volume of the oral cavity as the pigs increased in size. At 16 weeks, no significant differences in cycle or swallow frequencies between groups were observed, demonstrating that transition strategies do not alter drinking behavior at the sequence level.

**FIGURE 2:**
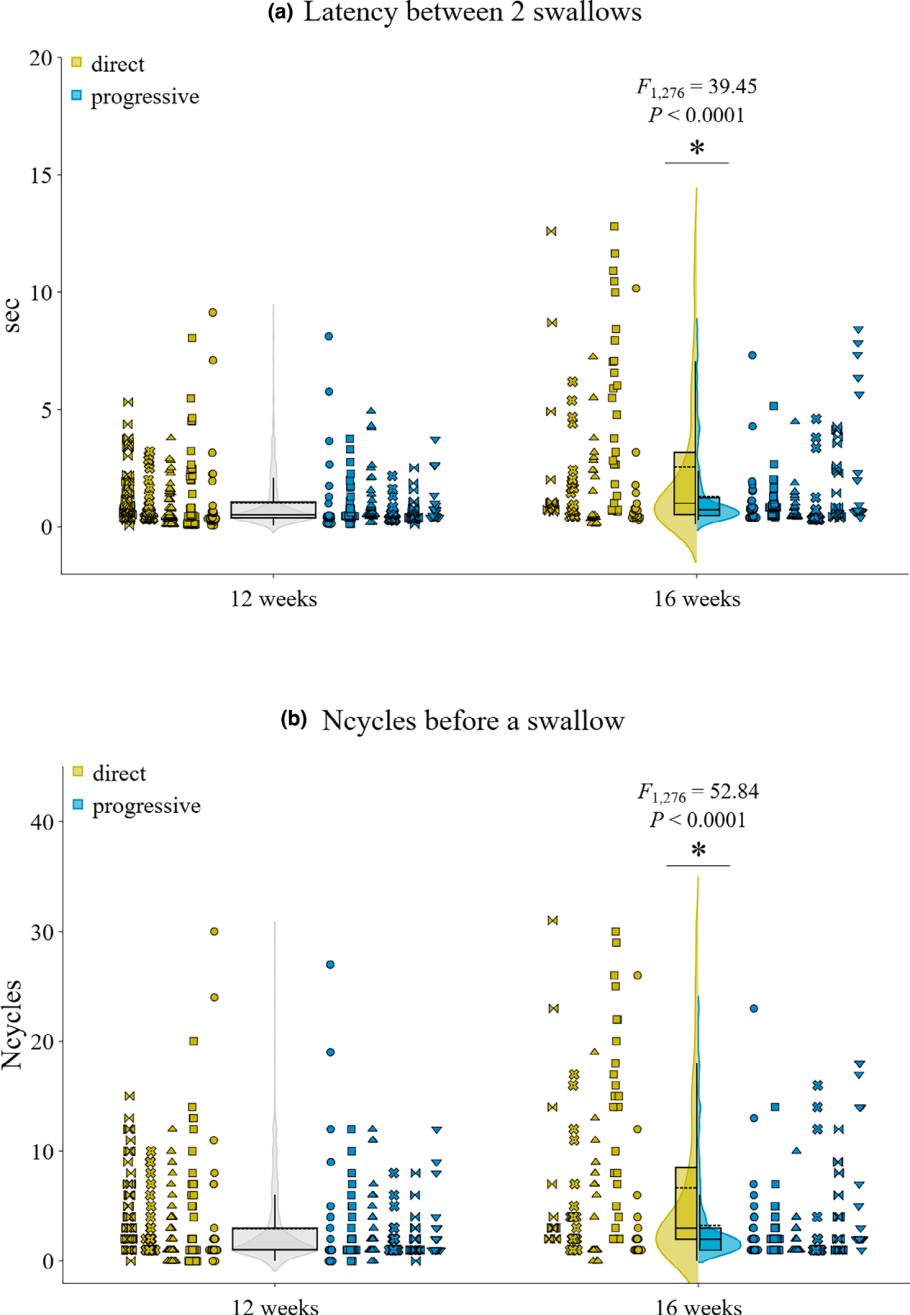
Effects of direct versus progressive transition strategy on swallowing behaviors during drinking in pigs: (a) Latency between 2 swallows (in seconds, sec), and (b) Number of cycles before a swallow. Colors indicate 16‐week transition groups: Direct transition (yellow) and progressive transition (blue). Data at 12 weeks of age are also presented (gray). For each group, violins illustrate data distribution, top and bottom of the boxplots represent the 1st (25th percentile) and 3rd (75th percentile) quartiles, respectively, and include the median (continuous line), mean (dash line), and whiskers (×1.5 interquartile range). Symbols represent individuals. Detailed individual averages and between‐individuals differences are provided in Supplemental Table S1.

At 12 weeks, pigs swallowed approximately once every second, which corresponds to approximately 1 swallow every 3 gape cycles (Table 2). After 16 weeks, both groups were characterized by more gape cycles intercalated between swallows, and accordingly, latency between swallows also increased (Table 2). Both the increase in latency and number of gape cycles before a swallow were significantly greater in the DT group than in the PT group (Figure 2), with a moderate to large effect size (Table S1).

### 3.2 Gape cycle data

Our results revealed that pigs usually swallowed liquids during an ingestion cycle (Table 2), i.e., while the tip of the tongue is outside of the oral cavity and in contact with the liquid in the bowl. In 12‐week old pigs, 452 of the 474 swallows recorded (95.4%) occurred during ingestion cycles, and swallows during transport cycles were rarely observed (4.6%). After 16 weeks, the majority of swallows still occurred during ingestion cycles, albeit the trend differed between the two groups. In the PT group 100% of the 174 swallows were initiated during ingestion cycles and there were no transport‐initiated swallows. In comparison, of the 119 swallows recorded in the DT group, the percentage of swallows initiated during ingestion decreased to 88.2%, with the remaining 11.8% initiated during transport cycles. These proportions mirror the fact that the majority of drinking cycles are ingestion cycles, with transport cycles only accounting for 17% and 3% of the total number of drinking cycles in the DT and PT group, respectively (Table 2).

On average, the presence of a swallow within a gape cycle increased the duration of that cycle, regardless of the initial behavior (Table 2). Moreover, transport cycles, with or without a swallow, were typically longer than ingestion cycles. In both transition groups, ingestion cycles during which a swallow occurs typically increased in duration compared to 12 weeks, but there were no differences between the 16‐week groups.

### 3.3 Epiglottal movements

**FIGURE 3:**
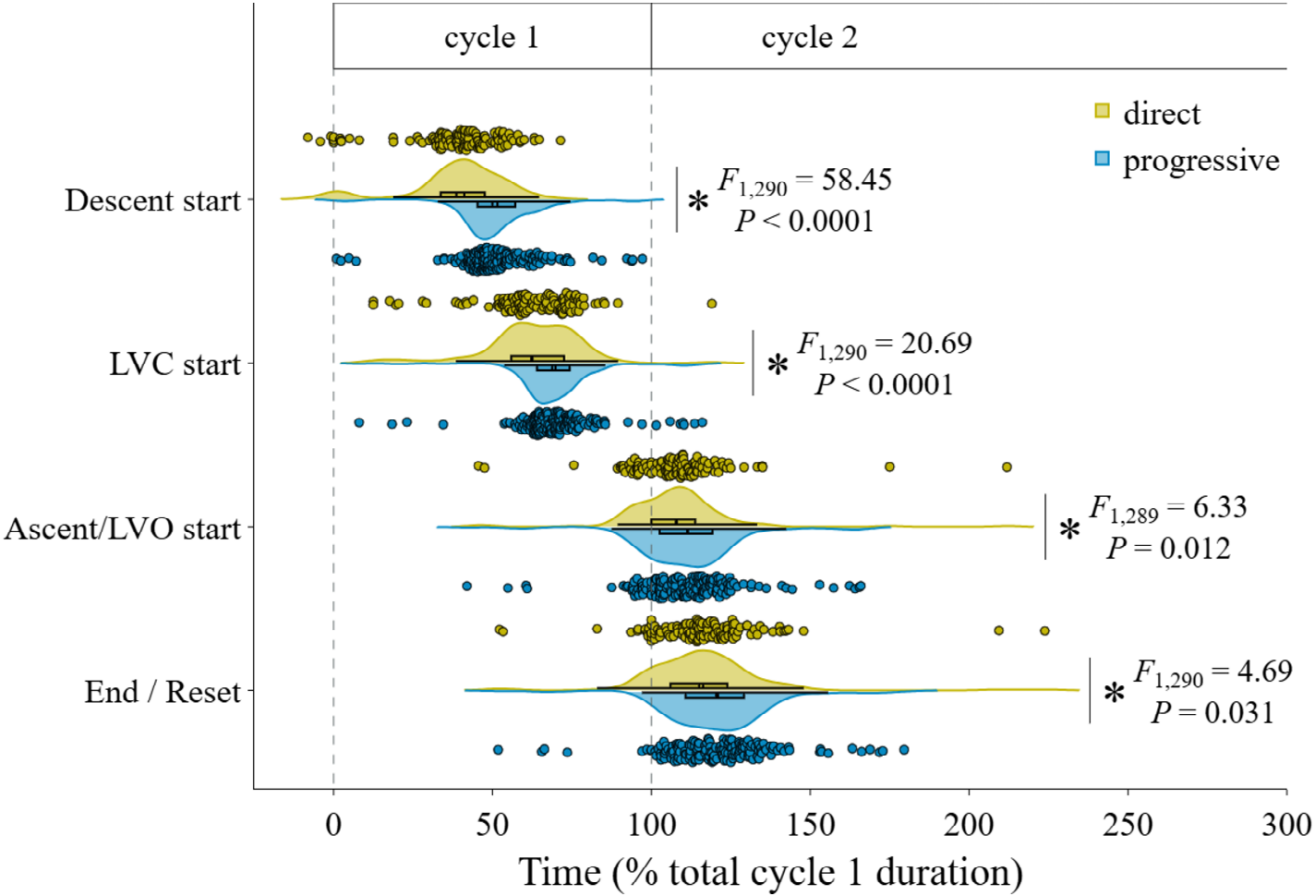
Effect of direct versus progressive transition strategy on the timing of epiglottal movements during liquid swallowing in 16‐week old pigs. Four timepoints were extracted and standardized as % time during the gape cycle duration in which the swallow was initiated (i.e., gape cycle 1): (1) start of epiglottal descent, (2) end of epiglottal descent, corresponding to the start to LVC, (3) start to epiglottal ascent and start to LVO (thus the end of LVC), and (4) end of epiglottal ascent, when the epiglottis resets to its rest position on the soft palate. Colors indicate transition group: Direct transition (yellow) and progressive transition (blue). Detailed individual averages and between‐individuals differences are provided in Table S4. Symbols represent individuals and asterisks indicate significant difference between the 2 groups.

The timing of 4 key epiglottis positions were standardized as a % of gape cycle duration to determine whether the occurrence of epiglottal movements shift within the gape cycle. A swallow was typically initiated around 50% of gape cycle duration and ended around 120%, indicating that the epiglottis resets to its resting position in the early stages of the next gape cycle (Table 3 and Figure 3). Epiglottis timings in 16‐week PT pigs more closely resemble the 12‐week pattern than DT pigs (Table 3). In 16‐week DT pigs, epiglottal descent started significantly earlier in the cycle compared to PT and 12‐week pigs, and this impacted the relative timing of each of the 3 subsequent key events (i.e., start to LVC, start to LVO, and time to epiglottis reset) in that all 3 occurred significantly earlier in the gape cycle in the DT group than in the PT group (Table 3 and Figure 3), with a moderate to large effect size (see Table S4). Note that bolus volume was a significant covariate for the first 2 timings (i.e., time to start and time to start to LVC), indicating that bigger bolus volumes were associated with delayed start of epiglottal movements, and delayed start to LVC (see Table S4). In contrast, neither time to start to LVO or time to the end of epiglottal ascent were correlated with bolus volume (see Table S4), likely because these movements occur when the bolus has already passed into the esophagus.

Over the course of the 4‐week experiment, the total duration of epiglottal movements (i.e., from start to reset) relative to gape cycle duration was affected differently by the treatment strategies in that swallows in PT pigs were significantly shorter than in DT pigs (Table 3). When comparing the relative duration of each epiglottal movement, the two groups are significantly different in descent and LVC duration, but not in ascent duration (Table 3). Specifically, swallows in PT pigs were characterized by shorter epiglottal descent and shorter LVC compared to DT pigs. Bolus volume was a significant covariate for the relative duration of total epiglottal movements and of LVC, indicating that bigger bolus volumes are positively correlated with longer LVC and longer total epiglottal movements (see Table S5). In contrast, the relative durations of epiglottal descent and ascent were not associated with changes in bolus volume as a covariate (see Table S5).

**FIGURE 4:**
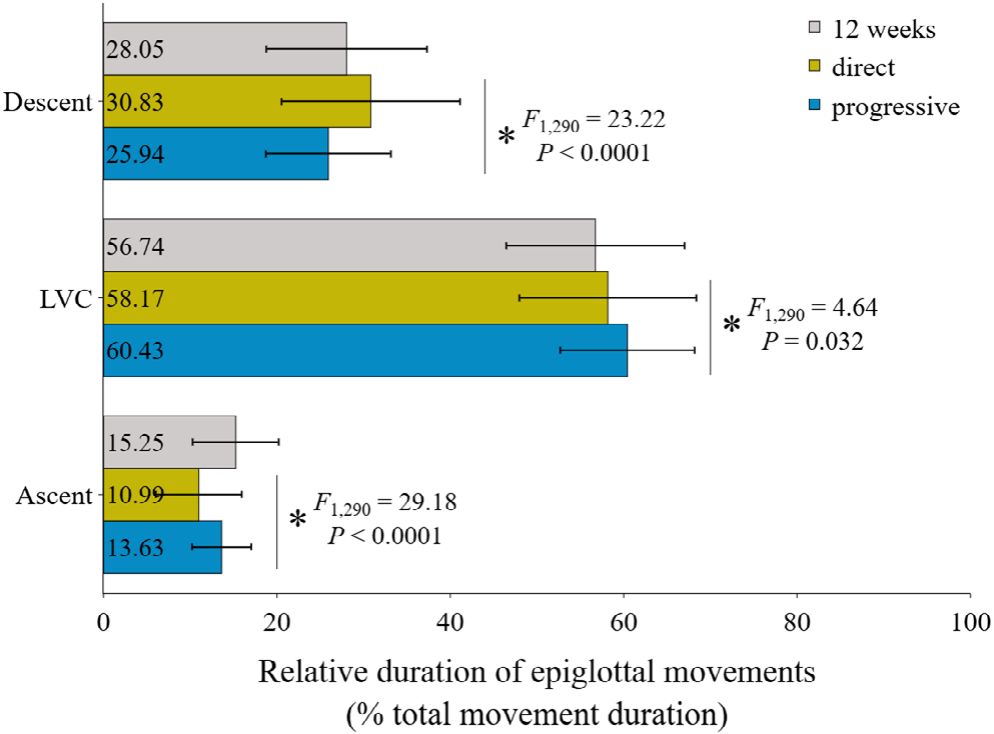
Relative durations (±standard deviation) of each stage of epiglottal movement (descent, LVC, ascent) as a % of total movement duration. Colors indicate groups: 12‐weeks (gray), 16‐week DT (yellow), and 16‐week PT (blue). Detailed individual averages and between‐individuals differences are provided in Table S6.

Finally, the durations of the 3 stages of epiglottal movement (i.e., descent, LVC, ascent) were standardized over their total combined duration in order to analyze their respective time allocation within the swallow. Compared to 12‐week data, both DT and PT groups increased standardized LVC duration, with the PT group demonstrating a statistically greater increase than the DT group (Figure 4 and Table S6). Both the PT and DT groups also decreased standardized ascent duration, with a significaly greater decrease in DT pigs than in PT pigs (Figure 4 and Table S6). In contrast, the standardized duration of epiglottal descent showed a different pattern of change in each group over the course of the 4‐week experiment. Indeed, compared to the 12‐week baseline, standardized epiglottal descent duration increased in the DT group and decreased in the PT group (Figure 4 and Table S6). The difference between the two transition groups at 16 weeks was statistically significant (Figure 4), with a moderate effect size (see Table S6). Note that bolus volume was not a significant covariate for the standardized durations of LVC or ascent, but was significant for descent standardized duration (see Table S6).

## 4 DISCUSSION

Food texture modification facilitates a safe transition from liquid ingestion at birth until solid foods can be adequately chewed and swallowed. During this developmental period, the infant demonstrates rapid anatomical, neuromotor, and sensorimotor maturation. Failure to acquire these skills through complementary feeding, which stimulates oral motor and sensory systems, has been anecdotally reported to negatively impact oromotor function and control, particularly during feeding as that is the therapeutic goal (Coulthard et al., 2009; da Costa et al., 2017; Northstone et al., 2001; Sdravou et al., 2023). As a result, it is important to examine how food texture transition strategies can affect swallowing, particularly any behaviors related to airway protection. To address a current critical gap in the literature related to the impact of textured modified diets in children, two groups of weaned pigs were transitioned from a liquidized diet to solid foods either directly or progressively using the IDDSI framework for texture modification of solids.

The objective of food texture modification is to facilitate the acquisition of motor skills needed to swallow solid foods safely, so neither transition strategy was anticipated to impact liquid swallowing behavior. As expected, the two transition groups were very similar in their overall drinking behavior. At 12 and 16 weeks, pigs in both groups were observed to swallow more often during an ingestion cycle (i.e., when the tongue made contact with the liquid in the bowl) than during transport (see Table 2). Thus the transition strategies had no impact on the overall pattern of when swallows occurred within the drinking sequence. This robust oromotor pattern reflects that applying moving stimuli to the oropharyngeal mucosa is more likely to evoke a swallow than stationary stimuli (Miller, 2002). Moreover, the swallows observed in the present study are typical for that age with the liquid passing “over a downturned epiglottis”, and reflect the “juvenile” swallow pattern described by Crompton et al. (2008). The general timing of swallowing in our animals was also comparable to those observed previously (Nakamura et al., n.d.; Hiiemae & Crompton, 1985; Thexton et al., 1998; Thexton & McGarrick, 1988), specifically cycle frequency and average swallowing frequency (see Table 2). For example, Thexton et al. (1998) found that swallows in typically weaned 3‐week old pigs “occurred within every third or fourth drinking cycle” (p.331). Similarly, Olson et al. (2021) reported drinking cycle duration in typically weaned 12‐week‐old pigs of ~280 msec, corresponding to 3.5 cycles per sec.

In addition to similarities at the sequence level, we found similarities at the cycle level. The swallow increased the duration of the gape cycle in which it was initiated, but did not interrupt the cyclical nature of jaw opening and closing during drinking. Once the liquid bolus was accumulated in the valleculae, swallows were typically initiated midway through the gape cycle when the mouth was closed (see Figure 3). In accordance with previous research (Thexton et al., 1998), as the gape cycle progressed, the epiglottis inverted to close the laryngeal vestibule (LVC), which is critical for airway protection. The swallow was then completed when the epiglottis returned to its resting position on the soft palate shortly after maximum gape during the early closing stages of the subsequent cycle.

However, our study revealed important differences in swallowing behavior between the progressive (PT) versus direct transition (DT) at 16 weeks. At the sequence level, PT pigs were characterized with fewer cycles and less time between swallows compared to the DT group (see Table 2 and Figure 2). In fact, swallow latency in PT pigs (1.3 s and 3 cycles) was more similar to the 12‐week pattern (1 s and 3 cycles), than DT pigs (>2 s and >6 cycles) (Table 2). Therefore, PT pigs did not increase swallow latency (either in time or in cycles) as much as DT pigs despite increasing in size between 12 and 16 weeks. Shorter latency between 2 swallows may be due to a number of factors including intrinsic parameters (e.g., anatomical characteristics of the oropharynx, ingestion efficiency, improved sensory processing), and bolus characteristics (e.g., volume, temperature, texture). At the anatomical level, animals with smaller valleculae can be expected to fill more rapidly before triggering the swallow (German et al., 2004), but in our dataset, the body size between groups at 16 weeks was not significant (see Table 1), therefore we can assume that the size of the valleculae was similar between groups.

Bolus volume was a significant covariate for swallow latency, with bigger boluses associated with longer latency between two consecutive swallows. On the other hand, bolus volume was not significantly different between the two treatment strategies at 16 weeks (see Table 2). Given these similarities, another hypothesis for the shorter latency is that PT pigs accumulate the volume necessary to trigger a swallow in less time than DT pigs. Taking into account that volume alone does not stimulate the swallow (German et al., 2004), shorter latency could also be interpreted as improved oral perception or processing of the liquid bolus prior to the swallow in PT pigs. Even though the data presented here do not provide explicit or unequivocal support for one hypothesis over another at this stage, our results reveal that food texture transition strategies impact the oropharyngeal phase of the liquid swallow.

At the level of the gape cycle, DT pigs were also observed to start the swallow significantly earlier in the gape cycle compared to PT pigs (39% vs. 52%, respectively; see Table 3 and Figure 3). Previously, Olson et al. (2021) reported jaw closing‐opening transition at 43% of the gape cycle during drinking from a bowl in typically‐weaned pigs of the same age, although in that study non‐swallowing and swallowing cycles were included. Using 43% as a benchmark for demarcating the drinking cycle between jaw closing and jaw opening, our results show that epiglottal descent in DT pigs was initiated around minimum gape during the late stage of jaw closing. In contrast, epiglottal descent in PT pigs started significantly later after minimum gape during the early stage of jaw opening (Table 3 and Figure 3). Interestingly, Thexton et al. (1998) also observed that the liquid swallow in juvenile 3‐week‐old pigs “was embedded in the early jaw‐opening phase” (p.327). Therefore, PT pigs appear to initiate the swallow at a similar timepoint as younger pigs and may have retained the juvenile pattern of swallow initiation. This underlying difference in the timing of swallow initiation suggests that transition strategies can affect the overall coordination between epiglottic movements and rhythmic jaw movements during drinking in pigs.

The total duration of epiglottal movement relative to gape cycle duration was significantly different between the 2 treatment strategies, with swallows in PT pigs being significantly shorter in duration than in DT pigs (see Table 3). Moreover, compared to the 12‐week baseline, our data show that the relative duration of epiglottal movement increased in DT pigs but decreased in PT pigs (see Table 3), thus revealing transition strategies have different effects on the maturation of a key component of the swallow. The durations of each epiglottal movement during swallowing (i.e, descent, LVC and ascent) are affected by transition strategies in different ways. In particular, the relative duration of epiglottal descent duration and LVC were both significantly shorter in the PT group compared to the DT group at 16 weeks of age, whereas ascent relative duration was similar (see Table 3). This reflects how the 2 stages of epiglottal movement that are arguably most important for airway protection (i.e., descent and LVC) are the most affected by transition strategies. Assuming similarities in the amount of epiglottal descent needed to reach LVC, a shorter descent duration implies that the epiglottis moves faster in PT pigs than in DT pigs. Furthermore, compared to 12 weeks, 16‐week old PT pigs decreased relative descent duration (from 28% to less than 26%), whereas DT pigs increased descent duration (from 28% to more than 30%; see Figure 4). This critical result highlights how each transition strategy altered descent duration in distinct ways throughout the course of the study. This study is the first to document how altering the consistency of foods during the transition to solids differentially influences the maturation of a key component of the swallow, and emphasizes how the transition to solid food is a critical period for swallow physiology development during liquid ingestion.

Compared to the DT group, epiglottal movement during the swallows in the PT group is characterized by shorter descent duration and longer LVC relative to the total duration of the swallow (see Figure 4), suggesting a tradeoff in duration between epiglottal descent and LVC within the context of the swallow. These results indicate that reducing epiglottal descent duration has a positive impact on LVC, and therefore, airway protection. Epiglottal descent and LVC are primarily passive movements that occur as the result of hyolaryngeal elevation and tongue base retraction (Logemann et al., 1992; Pearson et al., 2016; Vose & Humbert, 2019). The tradeoff between epiglottal descent and LVC relative to swallow duration suggests that the observed differences between DT and PT pigs could translate in differences in hyolaryngeal excursion and pharyngeal pressures as well, with PT pigs predicted to have greater excursion and pressures. In addition, we would predict differences in the ability to recruit and sustain the activation of suprahyoid, longitudinal pharyngeal, and the tongue base retractor muscles, hyoglossus and styloglossus (Gassert & Pearson, 2016; Pearson et al., 2012). Prolonged LVC duration relative to the swallow may also reflect that PT pigs exert increased effort to swallow liquids, especially given the fact that bolus volume was not significantly between the two treatment strategies. At this point, it is unclear whether prolonged LVC is a compensatory strategy or a fundamental change in the neurophysiology underlying the biomechanical events leading to greater airway protection.

We acknowledge one caveat to this study and suggest areas of future research. Previously, data on the development of swallowing in pigs focused on experimental conditions associated with infant oropharyngeal behaviors, where both the volume swallowed and the rate of swallowing are constrained (i.e., suckling out of a nipple bottle). Because the ability to control and swallow unregulated bolus volume (i.e., open cup drinking) is a critical next step in development and maturation of safe swallows, our study was designed to allow pigs to drink and swallow at their own pace. Accordingly, offering the liquid in a bowl was the best approach to keep the animal in the field of view of the X‐ray cameras to record the entirety of the drinking sequence, with all the intercalated swallows, while maintaining the most naturalistic environment. The next objectives of our project are to quantify hyolaryngeal, tongue, and epiglottal kinematics directly (e.g., amplitude and velocity of movements) in order to further our understanding of the underlying factors and mechanisms driving the differences observed in swallowing behaviors in juvenile pigs that are still maturing to reach the full adult patterns.

## 5 CONCLUSIONS

In spite of many overall similarities in the behavioral aspects of liquid swallowing, our study demonstrates that the transition strategies significantly alter critical characteristics of liquid swallowing in pigs, including the duration and timing of liquid swallow initiation, as well as the duration of key epiglottal movements, especially laryngeal vestibule closure which corresponds to airway protection. These findings expand our understanding of liquid swallowing behavior in mammals, and emphasize how texture modification strategies used to transition to solids can have unexpected impacts on managing liquid boluses. While neither transition group was negatively impacted in terms of airway protection, the observed differences suggest potentially important differences in the coordination of the oral and pharyngeal components of the swallow.

## AUTHOR CONTRIBUTIONS

**Stephane J. Montuelle:** Conceptualization; data curation; formal analysis; investigation; methodology; project administration; resources; software; supervision; validation; visualization. **Donna R. Scarborough:** Conceptualization; funding acquisition; investigation; methodology; project administration; resources; supervision; validation. **Hannah R. Baker:** Formal analysis; investigation. **Susan H. Williams:** Conceptualization; funding acquisition; investigation; methodology; project administration; resources; supervision; validation.

## FUNDING INFORMATION

This project was funded by the National Institutes of Health R21HD108761.

## CONFLICTS OF INTEREST STATEMENT

The authors do not have any conflicts of interest to report.

## ETHICS STATEMENT

All procedures and experiments involving live animals were monitored and approved by the Ohio University Institutional Animal Care and Use Committee (protocol #22‐U‐001). All the sequences recorded for each individual were included in the final analysis. To minimize analytical bias, individual IDs were randomly assigned within each treatment group, and video sequences were only identified with individual ID, with no reference to their treatment group, thereby ensuring that data analysis was carried out blind. The authors did not use AI tools at any stage of figure, table and manuscript preparation.

## Supporting information


**Table S1:** Key behavioral characteristics of drinking behavior and swallowing in 12‐ and 16‐week old pigs. Table entries are group means (bold) and individual average ± standard deviation. For each variable, the effect of the transition strategy (direct versus progressive) was assessed by testing whether the between‐group difference was significant. Between‐individual differences were also tested, and bolus volume was included as a covariate for the swallow‐level variables (i.e., swallow latency and Ncycles before a swallow).
**Table S2:** Proportion of liquid swallows occurring during an ingestion or a transport gape cycle in 12‐ and 16‐week old pigs for each transition strategy. The average proportion of each behavior throughout the entire drinking sequence is also presented. Table entries are group means (bold) and individual average ± standard deviation.
**Table S3:** Cycle duration with and without a swallow during drinking behavior in 12‐ and 16‐week old pigs. Table entries are group means (bold) and individual average ± standard deviation. For each variable, the effect of the transition strategy (direct versus progressive) was assessed by testing whether the between‐group difference was significant. Between‐individual differences were also tested, and bolus volume was included as a covariate for the swallow‐level variables (i.e., swallow cycle duration).
**Table S4:** Epiglottal movement timings during drinking behavior in 12‐ and 16‐week old pigs. Time is standardized to % of gape cycle duration. Table entries are group means (bold) and individual average ± standard deviation. For each variable, the effect of the transition strategy (direct versus progressive) was assessed by testing whether the between‐group difference was significant. Bolus volume was included as a covariate, and between‐individual differences were also tested.
**Table S5:** Epiglottal movement durations during drinking behavior in 12‐ and 16‐week old pigs. Durations are presented in absolute values (msec) and as % total gape cycle duration. Table entries are group means (bold) and individual average ± standard deviation. For each variable, the effect of the transition strategy (direct versus progressive) was assessed by testing whether the between‐group difference was significant. Bolus volume was included as a covariate, and between‐individual differences were also tested.
**Table S6:** Relative duration of each phase of epiglottal inversion (descent, laryngeal vestibule closure LVC, ascent) during drinking behavior in 12‐ and 16‐week old pigs. Table entries are group means (bold) and individual average ± standard deviation. For each variable, the effect of the transition strategy (direct versus progressive) was assessed by testing whether the between‐group difference was significant. Bolus volume was included as a covariate, and between‐individual differences were also tested.

## Data Availability

Data used in statistical analyses is available upon request.
