# Supplementary material for: Transitional feeding in developing pigs: Effects of food texture modification on liquid swallowing behavior and epiglottic movements
Source: Physiol Rep. 2026 Apr 17;14(8):e70878. doi: 10.14814/phy2.70878 (PMC13090537; doi:10.14814/phy2.70878)
Supplement: Supplementary file 1 — Table S1: Key behavioral characteristics of drinking behavior and swallowing in 12‐ and 16‐week old pigs. Table entries are group means (bold) and individual average ± standard deviation. For each variable, the effect of the transition strategy (direct versus progressive) was assessed by testing whether the between‐group difference was significant. Between‐individual differences were also tested, and bolus volume was included as a covariate for the swallow‐level variables (i.e., swallow latency and Ncycles before a swallow). Table S2: Proportion of liquid swallows occurring during an ingestion or a transport gape cycle in 12‐ and 16‐week old pigs for each transition strategy. The average proportion of each behavior throughout the entire drinking sequence is also presented. Table entries are group means (bold) and individual average ± standard deviation. Table S3: Cycle duration with and without a swallow during drinking behavior in 12‐ and 16‐week old pigs. Table entries are group means (bold) and individual average ± standard deviation. For each variable, the effect of the transition strategy (direct versus progressive) was assessed by testing whether the between‐group difference was significant. Between‐individual differences were also tested, and bolus volume was included as a covariate for the swallow‐level variables (i.e., swallow cycle duration). Table S4: Epiglottal movement timings during drinking behavior in 12‐ and 16‐week old pigs. Time is standardized to % of gape cycle duration. Table entries are group means (bold) and individual average ± standard deviation. For each variable, the effect of the transition strategy (direct versus progressive) was assessed by testing whether the between‐group difference was significant. Bolus volume was included as a covariate, and between‐individual differences were also tested. Table S5: Epiglottal movement durations during drinking behavior in 12‐ and 16‐week old pigs. Durations are presented in absolute values (msec) [file PHY2-14-e70878-s001.docx]

**Montuelle et al – Manuscript ID PHYSREP-2026-01-031-T**

***Transitional feeding in developing pigs: effects of food texture modification on liquid swallowing behavior and epiglottic movements***

**SUPPLEMENTAL TABLES - S1 through S6**

**Table S1.** Key behavioral characteristics of drinking behavior and swallowing in 12- and 16-week old pigs. Table entries are group means (bold) and individual average ± standard deviation. For each variable, the effect of the transition strategy (direct versus progressive) was assessed by testing whether the between-group difference was significant. Between-individual differences were also tested, and bolus volume was included as a covariate for the swallow-level variables (i.e., swallow latency and Ncycles before a swallow).

| **Variable** | **12 weeks** | | **16 weeks** | |
| --- | --- | --- | --- | --- |
|  | **All individuals** | | **DT** | **PT** |
| **Cycle Frequency**  cycles per sec | **3.20 ± 0.26** | | **2.71 ± 0.23** | **2.89 ± 0.38** |
|  | 43: 3.44  48: 2.77 ± 0.09  53: 3.10 ± 0.06  54: 3.14 ± 0.04  55: 3.27 ± 0.24 | 40: 3.27  45: 2.97  49: 2.71  52: 3.77  56: 3.34 ± 0.19  57: 3.23 ± 0.07 | 43: 3.06  48: 2.51  53: 2.78  54: 2.76  55: 2.81 | 40: 3.19  45: 2.83  49: 2.64  52: 3.31  56: 2.38  57: 2.58 |
|  | Diet: Non-significant (*P* = 0.391), Effect size 𝜂^2^ = 0.11  Individual: Non-significant (*P* = 0.056) | | Diet: Non-significant (*P* = 0.296), Effect size 𝜂^2^ = 0.09  Individual(DT): Non-significant (P = 0.289)  Individual(PT): Non-significant (P = 0.128) | |
| **Swallow Frequency**  swallows per sec | **0.76 ± 0.35** | | **0.35 ± 0.20** | **0.53 ± 0.25** |
|  | 43: 0.62  48: 0.45 ± 0.10  53: 0.77 ± 0.16  54: 0.75 ± 0.36  55: 0.57 ± 0.32 | 40: 0.44  45: 0.86  49: 0.66  52: 1.24  56: 1.05 ± 0.41  57: 0.81 ± 0.55 | 43: 0.63  48: 0.17  53: 0.53  54: 0.45  55: 0.32 | 40: 0.67  45: 0.75  49: 0.71  52: 0.39  56: 0.45  57: 0.36 |
|  | Diet: Non-significant (*P* = 0.057), Effect size 𝜂^2^ = 0.15  Individual: Non-significant (*P* = 0.990) | | Diet: Non-significant (*P* = 0.155), Effect size 𝜂^2^ = 0.16  Individual(DT): Non-significant (P = 0.160)  Individual(PT): Non-significant (P = 0.998) | |
| **Bolus volume**  mL | **8.6 ± 9.4** | | **10.8 ± 11.1** | **9.2 ± 11.0** |
|  | 43: 7.3 ± 11.7  48: 6.5 ± 9.4  53: 7.9 ± 10.2  54: 11.2 ± 10.8  55: 10.5 ± 8.3 | 40: 7.6 ± 10.7  45: 5.9 ± 7.0  49: 5.9 ± 7.4  52: 5.8 ± 4.0  56: 11.9 ± 8.8  57: 12.8 ± 12.2 | 43: 10.4 ± 7.2  48: 7.3 ± 4.1  53: 12.0 ± 13.0  54: 10.7 ± 9.9  55: 15.7 ± 18.4 | 40: 11.6 ± 14.6  45: 6.7 ± 6.4  49: 6.0 ± 6.3  52: 7.7 ± 11.4  56: 10.8 ± 9.3  57: 15.4 ± 16.1 |
|  | Diet: Non-significant (*P* = 0.744), Effect size 𝜂^2^ = 0.01  Individual: *P* = 0.002 | | Diet: Non-significant (*P* = 0.196), Effect size 𝜂^2^ = 5.8e-3  Individual(DT): Non-significant (P = 0.076)  Individual(PT): P = 0.037 | |
| **Latency between 2 swallows**  sec | **1.09 ± 1.58** | | **2.54 ± 3.07** | **1.28 ± 1.52** |
|  | 43: 1.09 ± 1.93  48: 1.75 ± 2.75  53: 1.00 ± 1.68  54: 0.89 ± 0.85  55: 1.37 ± 1.21 | 40: 1.73 ± 3.09  45: 0.83 ± 0.81  49: 1.14 ± 1.19  52: 0.55 ± 0.40  56: 0.63 ± 0.48  57: 0.95 ± 0.91 | 43: 1.29 ± 2.14  48: 5.09 ± 3.79  53: 1.61 ± 1.84  54: 1.75 ± 1.63  55: 2.28 ± 3.26 | 40: 1.27 ± 1.46  45: 1.08 ± 0.87  49: 0.94 ± 0.90  52: 1.03 ± 1.49  56: 1.69 ± 1.46  57: 2.38 ± 2.80 |
|  | Diet: Non-significant (*P* = 0.539), Effect size 𝜂^2^ = 0.04  Bolus volume: *F*_1,440_ = 682.55, *P* < 0.0001  Individual: *P* < 0.0001 | | Diet: *F*_1,276_ = 39.45, *P* < 0.0001, Effect size 𝜂^2^ = 0.13  Bolus volume: *F*_1,276_ = 250.10, *P* < 0.0001  Individual(DT): *P* < 0.0001; Individual(PT): *P* = 0.007 | |
| **Number of Cycles before a swallow** | **3.2 ± 4.9** | | **6.7 ± 7.6** | **3.2 ± 3.9** |
|  | 43: 3.6 ± 6.5  48: 4.6 ± 7.6  53: 2.9 ± 5.5  54: 2.6 ± 2.6  55: 4.5 ± 3.7 | 40: 5.4 ± 10.2  45: 2.2 ± 2.6  49: 2.8 ± 3.1  52: 1.9 ± 1.5  56: 1.8 ± 1.5  57: 2.8 ± 3.1 | 43: 3.7 ± 5.9  48: 13.2 ± 8.9  53: 4.3 ± 5.0  54: 4.6 ± 4.6  55: 6.5 ± 8.4 | 40: 3.6 ± 4.7  45: 2.5 ± 2.2  49: 2.0 ± 1.9  52: 2.7 ± 4.2  56: 3.8 ± 3.2  57: 6.0 ± 6.3 |
|  | Diet: Non-significant (*P* = 0.499), Effect size 𝜂^2^ = 0.05  Bolus volume: *F*_1,440_ = 902.24, *P* < 0.0001  Individual: *P* < 0.0001 | | Diet: *F*_1,276_ = 52.84, *P* < 0.0001, Effect size 𝜂^2^ = 0.16  Bolus volume: *F*_1,276_ = 322.13, *P* < 0.0001  Individual(DT): *P* < 0.0001  Individual(PT): *P* = 0.035 | |

**Table S2.** Proportion of liquid swallows occurring during an ingestion or a transport gape cycle in 12- and 16-week old pigs for each transition strategy. The average proportion of each behavior throughout the entire drinking sequence is also presented. Table entries are group means (bold) and individual average ± standard deviation.

| **Variable** | **12 weeks** | | **16 weeks** | |
| --- | --- | --- | --- | --- |
|  | **All individuals** | | **DT** | **PT** |
| **%** **Ingestion swallows** | **95.4%** | | **88.2%** | **100%** |
|  | 43: 100% (35/35)  48: 90.9% (40/44)  53: 100% (61/61)  54: 98.0% (48/49)  55: 96.0% (72/75) | 40: 92.3% (24/26)  45: 97.8% (44/45)  49: 70.3% (26/37)  52: 100% (37/37)  56: 100% (42/42)  57: 100% (23/23) | 43: 90.5% (19/21)  48: 59% (17/29)  53: 100% (25/25)  54: 100% (26/26)  55: 100% (18/18) | 40: 100% (27/27)  45: 100% (43/43)  49: 100% (28/28)  52: 100% (29/29)  56: 100% (26/26)  57: 100% (21/21) |
| **% Transport swallows** | **4.6%** | | **11.8%** | **0%** |
|  | 43: absent (0/35)  48: 9.3% (4/44)  53: absent (0/60)  54: 2.0% (1/49)  55: 4.0% (3/75) | 40: 7.7% (2/26)  45: 2.2% (1/45)  49: 29.7% (11/37)  52: absent (0/37)  56: absent (0/42)  57: absent (0/23) | 43: 9.5% (2/21)  48: 41% (12/29)  53: absent (0/25)  54: absent (0/26)  55: absent (0/18) | 40: absent (0/27)  45: absent (0/43)  49: absent (0/28)  52: absent (0/29)  56: absent (0/26)  57: absent (0/21) |
| **Ingestion/Transport cycle ratio** | **89.0% ingestion**  **11.0% transport** | | **82.6% ingestion**  **17.4% transport** | **96.8% ingestion**  **3.2% transport** |
|  | 43: 90% ; 10%  45: 97% ; 3%  48: 71% ; 29%  53: 93% ; 7%  54: 95% ; 5%  55: 98% ; 2% | 40: 83% ; 17%  49: 63% ; 37%  52: 96% ; 4%  56: 100% ; 0%  57: 95% ; 5% | 43: 67% ; 33%  48: 74% ; 26%  53: 92% ; 8%  54: 98% ; 2%  55: 96% ; 4% | 40: 100% ; 0%  45: 100% ; 0%  49: 90% ; 10%  52: 92% ; 8%  56: 98% ; 2%  57: 100% ; 0% |

**Table S3**. Cycle duration with and without a swallow during drinking behavior in 12- and 16-week old pigs. Table entries are group means (bold) and individual average ± standard deviation. For each variable, the effect of the transition strategy (direct versus progressive) was assessed by testing whether the between-group difference was significant. Between-individual differences were also tested, and bolus volume was included as a covariate for the swallow-level variables (i.e., swallow cycle duration).

| **Variable** | **12 weeks** | | **16 weeks** | |
| --- | --- | --- | --- | --- |
|  | **All individuals** | | **DT** | **PT** |
| **Ingestion swallow Cycle Duration**  msec | **329 ± 50** | | **365 ± 94** | **364 ± 66** |
|  | 43: 308 ± 44  48: 362 ± 40  53: 338 ± 40  54: 332 ± 42  55: 338 ± 46 | 40: 334 ± 33  45: 281 ± 28  49: 395 ± 45  52: 280 ± 20  56: 324 ± 52  57: 339 ± 42 | 43: 316 ± 43  48: 373 ± 81  53: 362 ± 74  54: 398 ± 132  55: 368 ± 89 | 40: 348 ± 56  45: 370 ± 29  49: 364 ± 54  52: 322 ± 54  56: 416 ± 90  57: 368 ± 83 |
|  | Diet: Non-significant (*P* = 0.632), Effect size 𝜂^2^ = 0.03  Bolus volume: *F*_1,441_ = 11.78, *P* = 0.0006  Individual: *P* < 0.0001 | | Diet: Non-significant (*P* = 0.907), Effect size 𝜂^2^ = 4.9e-5  Bolus volume: *F*_1,276_ = 22.48, *P* < 0.0001  Individual(DT): Non-significant (P = 0.182)  Individual(PT): P < 0.0001 | |
| **Ingestion Cycle Duration**  msec | **298 ± 56** | | **361 ± 100** | **350 ± 102** |
|  | 43: 282 ± 54  48: 330 ± 56  53: 316 ± 66  54: 304 ± 38  55: 291 ± 48 | 40: 297 ± 50  45: 255 ± 25  49: 361 ± 48  52: 256 ± 26  56: 295 ± 67  57: 296 ± 52 | 43: 293 ± 54  48: 379 ± 112  53: 360 ± 95  54: 354 ± 89  55: 352 ± 84 | 40: 304 ± 40  45: 348 ± 41  49: 372 ± 91  52: 285 ± 59  56: 417 ± 147  57: 390 ± 113 |
|  | Diet: Non-significant (*P* = 0.572), Effect size 𝜂^2^ = 0.04  Individual: *P* < 0.0001 | | Non-significant (*P* = 0.058), Effect size 𝜂2 = 2.6e-3  Individual(DT): *P* < 0.0001, Individual(PT): *P* < 0.0001 | |
| **Transport swallow Cycle Duration**  msec | **398 ± 79** | | **485 ± 41** | **NA - absent** |
|  | 43: NA - absent  48: 456 ± 44  53: NA - absent  54: 415  55: 380 ± 104 | 40: 320 ± 14  45: 240  49: 409 ± 70  52: NA - absent  56: NA - absent  57: NA - absent | 43: 410 ± 64  48: 497 ± 22  53: NA - absent  54: NA - absent  55: NA - absent | 40: NA - absent  45: NA - absent  49: NA - absent  52: NA - absent  56: NA - absent  57: NA - absent |
|  | Diet: Non-significant (*P* = 0.305), Effect size 𝜂^2^ = 0.06  Bolus volume: Non-significant (*P* = 0.311)  Individual: Non-significant (*P* = 0.082) | | NA | |
| **Transport Cycle Duration**  msec | **364 ± 70** | | **417 ± 89** | **427 ± 99** |
|  | 43: 324 ± 51  48: 407 ± 33  53: 344 ± 57  54: 401 ± 76  55: 347 ± 53 | 40: 318 ± 88  45: 326 ± 191  49: 358 ± 55  52: 287 ± 23  56: NA - absent  57: 359 ± 110 | 43: 336 ± 74  48: 450 ± 81  53: 354 ± 58  54: 404 ± 24  55: 394 ± 32 | 40: 470 ± 52  45: NA - absent  49: NA - absent  52: 374 ± 89  56: 545 ± 120  57: NA - absent |
|  | Diet: Non-significant (*P* = 0.171), Effect size 𝜂^2^ = 0.21  Individual: *P* < 0.0001 | | Non-significant (*P* = 0.613), Effect size 𝜂2 = 1.4e-3  Individual(DT): *P* < 0.0001, Individual(PT): *P* = 0.009 | |

**Table S4**. Epiglottal movement timings during drinking behavior in 12- and 16-week old pigs. Time is standardized to % of gape cycle duration. Table entries are group means (bold) and individual average ± standard deviation. For each variable, the effect of the transition strategy (direct versus progressive) was assessed by testing whether the between-group difference was significant. Bolus volume was included as a covariate, and between-individual differences were also tested.

| **Variable** | **12 weeks** | | **16 weeks** | |
| --- | --- | --- | --- | --- |
|  | **All individuals** | | **DT** | **PT** |
| **Time to the start of epiglottal descent**  % cycle duration | **49.0% ± 11.3** | | **38.7% ± 15.8** | **51.6% ± 13.1** |
|  | 43: 43.6% ± 9.4  48: 44.0% ± 9.2  53: 46.0% ± 11.3  54: 45.7% ± 7.5  55: 48.3% ± 13.1 | 40: 49.9% ± 8.1  45: 58.7% ± 7.8  49: 54.2% ± 8.0  52: 53.5% ± 8.4  56: 50.5% ± 10.0  57: 45.0% ± 18.0 | 43: 41.8% ± 9.8  48: 36.6% ± 11.2  53: 39.5% ± 19.3  54: 37.6% ± 20.5  55: 38.8% ± 15.4 | 40: 51.7% ± 9.7  45: 49.5% ± 5.1  49: 62.3% ± 15.1  52: 51.0% ± 7.2  56: 49.1% ± 13.9  57: 45.1% ± 21.7 |
|  | Diet: Non-significant (*P* = 0.077), Effect size 𝜂^2^ = 0.52  Bolus volume: *F*_1,466_ = 5.50, *P* = 0.019  Individual: *P* < 0.0001 | | Diet: *F*_1,290_ = 58.45, *P* < 0.0001, Effect size 𝜂^2^ = 0.17  Bolus volume: *F*_1,290_ = 4.18, *P* = 0.042,  Individual(DT): *P* = 0.998; Individual(PT): *P* = 0.0002 | |
| **Time to LVC / end of epiglottal descent**  % cycle duration | **70.2% ± 13.3** | | **62.2% ± 15.6** | **69.8% ± 13.1** |
|  | 43: 69.5% ± 8.3  48: 68.8% ± 10.9  53: 62.0% ± 10.1  54: 71.0% ± 12.0  43: 67.6% ± 15.8 | 40: 72.7% ± 11.2  45: 85.3% ± 14.7  49: 65.0% ± 8.7  52: 74.8% ± 8.3  56: 72.4% ± 8.7  57: 65.7% ± 18.4 | 43: 65.4% ± 9.7  48: 63.9% ± 14.9  53: 63.4% ± 16.1  54: 59.7% ± 19.3  55: 57.8% ± 15.7 | 40: 72.8% ± 7.3  45: 66.4% ± 6.0  49: 74.0% ± 16.2  52: 72.6% ± 9.7  56: 66.1% ± 13.6  57: 68.2% ± 23.0 |
|  | Diet: Non-significant (*P* = 0.211), Effect size 𝜂^2^ = 0.17  Bolus volume: *F*_1,438_ = 23.30, *P* < 0.0001  Individual: *P* < 0.0001 | | Diet: *F*_1,290_ = 20.69, *P* < 0.0001, Effect size 𝜂^2^ = 0.07  Bolus volume: *F*_1,290_ = 5.30, *P* = 0.022,  Individual(DT): Non-significant (*P* = 0.997); Individual(PT): Non-significant (*P* = 0.123) | |
| **Time to LVO / start of epiglottal ascent**  % cycle duration | **112.0% ± 13.9** | | **107.0% ± 14.4** | **112.0% ± 16.5** |
|  | 43: 112% ± 10.5  48: 104% ± 12.1  53: 107% ± 9.0  54: 110% ± 6.3  55: 113% ± 11.5 | 40: 108% ± 11.6  45: 130% ± 12.3  49: 107% ± 10.2  52: 115% ± 10.6  56: 116% ± 7.1  57: 104% ± 18.3 | 43: 107% ± 8.0  48: 99% ± 7.4  53: 111% ± 13.2  54: 109% ± 23.2  55: 110% ± 8.4 | 40: 109% ± 8.3  45: 103% ± 7.9  49: 122% ± 19.4  52: 120% ± 10.7  56: 107% ± 15.7  57: 114% ± 26.4 |
|  | Diet: Non-significant (*P* = 0.505), Effect size 𝜂^2^ = 0.05  Bolus volume: *F*_1,436_ = 21.50, *P* < 0.0001  Individual: *P* < 0.0001 | | Diet: *F*_1,289_ = 6.33, *P* = 0.012, Effect size 𝜂^2^ = 0.02  Bolus volume: Non-significant (*P* = 0.761)  Individual(DT): *P* = 0.028; Individual(PT): *P* < 0.0001 | |
| **Time to the end of epiglottal ascent**  % cycle duration | **124.0% ± 16.1** | | **116.0% ± 19.6** | **121.0% ± 17.2** |
|  | 43: 121% ± 11.9  48: 116% ± 15.1  53: 120% ± 13.0  54: 122% ± 7.1  55: 127% ± 18.3 | 40: 117% ± 12.4  45: 143% ± 12.9  49: 113% ± 10.3  52: 132% ± 10.6  56: 128% ± 17.5  57: 117% ± 18.9 | 43: 117% ± 9.7  48: 105% ± 8.6  53: 117% ± 13.2  54: 116% ± 21.3  55: 121% ± 10.6 | 40: 117% ± 9.1  45: 113% ± 8.8  49: 128% ± 20.3  52: 133% ± 11.1  56: 116% ± 17.1  57: 124% ± 26.7 |
|  | Diet: Non-significant (*P* = 0.487), Effect size 𝜂^2^ = 0.06  Bolus volume: *F*_1,464_ = 16.27, *P* < 0.0001  Individual: *P* < 0.0001 | | Diet: *F*_1,290_ = 4.69, *P* = 0.031, Effect size 𝜂^2^ = 0.02  Bolus volume: Non-significant (*P* = 0.746)  Individual(DT): Non-significant (*P* = 0.374) Individual(PT): *P* < 0.001 | |

**Table S5**. Epiglottal movement durations during drinking behavior in 12- and 16-week old pigs. Durations are presented in absolute values (msec) and as % total gape cycle duration. Table entries are group means (bold) and individual average ± standard deviation. For each variable, the effect of the transition strategy (direct versus progressive) was assessed by testing whether the between-group difference was significant. Bolus volume was included as a covariate, and between-individual differences were also tested.

| **Variable** | **12 weeks** | | **16 weeks** | |
| --- | --- | --- | --- | --- |
|  | **All individuals** | | **DT** | **PT** |
| **Total epiglottal movement duration**  % cycle duration | **74.8% ± 12.6** | | **77.6% ± 22.9** | **69.4% ± 12.9** |
|  | 43: 77.5% ± 9.8  48: 71.6% ± 12.2  53: 73.8% ± 13.2  54: 76.6% ± 8.1  55: 78.7% ± 13.4 | 40: 67.3% ± 10.4  45: 84.7% ± 12.6  49: 58.9% ± 6.8  52: 78.0% ± 7.1  56: 74.7% ± 11.2  57: 72.2% ± 10.2 | 43: 75.4% ± 6.9  48: 72.1% ± 22.2  53: 77.5% ± 21.7  54: 82.4% ± 32.3  55: 82.5% ± 21.4 | 40: 65.3% ± 7.5  45: 63.1% ± 7.2  49: 65.8% ± 12.0  52: 81.8% ± 12.6  56: 66.8% ± 14.5  57: 78.7% ± 11.3 |
|  | Diet: Non-significant (*P* = 0.505), Effect size 𝜂^2^ = 0.05  Bolus volume: *F*_1,464_ = 8.73, *P* = 0.003  Individual: *P* < 0.0001 | | Diet: *F*_1,290_ = 15.43, *P* = 0.0001, Effect size 𝜂^2^ = 0.05  Bolus volume: *F*_1,290_ = 3.96, *P* = 0.048  Individual(DT): Non-significant (*P* = 0.973)  Individual(PT): *P* < 0.0001 | |
| **Epiglottal descent duration**  % cycle duration | **21.1% ± 8.3** | | **23.6% ± 9.0** | **18.3% ± 7.1** |
|  | 43: 25.8% ± 6.5  48: 24.8% ± 6.6  53: 16.0% ± 4.9  54: 25.4% ± 8.7  55: 18.5% ± 5.3 | 40: 22.8% ± 7.7  45: 26.6% ± 11.6  49: 10.8% ± 4.0  52: 21.2% ± 5.4  56: 21.7% ± 6.1  57: 20.7% ± 5.5 | 43: 23.6% ± 5.8  48: 27.3% ± 9.3  53: 24.0% ± 10.0  54: 22.1% ± 10.0  55: 19.1% ± 5.9 | 40: 21.2% ± 5.0  45: 16.9% ± 4.0  49: 11.8% ± 5.3  52: 21.5% ± 10.1  56: 17.0% ± 6.0  57: 23.1% ± 5.3 |
|  | Diet: Non-significant (*P* = 0.650), Effect size 𝜂^2^ = 0.02  Bolus volume: *F*_1,437_ = 20.50, *P* < 0.0001  Individual: *P* < 0.0001 | | Diet: *F*_1,290_ = 31.70, *P* < 0.001, Effect size 𝜂^2^ = 0.10  Bolus volume: Non-significant (*P* = 0.669)  Individual(DT): Non-significant (*P* = 0.099) Individual(PT): *P* < 0.0001 | |
| **Laryngeal vestibule closure duration**  % cycle duration | **42.1% ± 9.2** | | **45.5% ± 17.3** | **41.7% ± 8.0** |
|  | 43: 42.6% ± 6.6  48: 35.0% ± 9.8  53: 45.5% ± 8.7  54: 39.3% ± 8.3  43: 49.3% ± 10.4 | 40: 35.0% ± 7.2  45: 45.1% ± 9.3  49: 41.5% ± 5.2  52: 40.3% ± 4.5  56: 43.2% ± 8.9  57: 38.8% ± 6.4 | 43: 41.4% ± 8.0  48: 38.7% ± 16.7  53: 47.6% ± 14.9  54: 49.7% ± 22.7  55: 52.1% ± 16.9 | 40: 35.9% ± 4.9  45: 36.3% ± 4.0  49: 47.6% ± 7.8  52: 47.8% ± 3.6  56: 40.6% ± 8.1  57: 45.3% ± 9.3 |
|  | Diet: Non-significant (*P* = 0.356), Effect size 𝜂^2^ = 0.04  Bolus volume: Non-significant (*P* = 0.798)  Individual: *P* < 0.0001 | | Diet: *F*_1,290_ = 6.43, *P* = 0.011, , Effect size 𝜂^2^ = 0.02  Bolus volume: *F*_1,290_ = 6.26, *P* = 0.013  Individual(DT): Non-significant (*P* = 0.101); Individual(PT): *P* < 0.0001 | |
| **Epiglottal ascent duration**  % cycle duration | **11.6% ± 5.0** | | **8.6% ± 4.9** | **9.5% ± 2.9** |
|  | 43: 9.1% ± 2.1  48: 12.0% ± 5.0  53: 12.3% ± 8.3  54: 12.0% ± 3.2  55: 12.1% ± 4.9 | 40: 9.3% ± 2.2  45: 13.0% ± 2.8  49: 6.5% ± 1.0  52: 16.5% ± 4.5  56: 9.7% ± 2.8  57: 12.7% ± 2.9 | 43: 10.5% ± 6.9  48: 6.1% ± 2.1  53: 5.9% ± 1.5  54: 10.7% ± 5.8  55: 11.2% ± 3.7 | 40: 8.3% ± 1.5  45: 10.0% ± 1.7  49: 6.4% ± 1.5  52: 12.5 % ± 2.5  56: 9.1% ± 4.1  57: 10.3% ± 1.8 |
|  | Diet: Non-significant (*P* = 0.916), Effect size 𝜂^2^ = 1.3e-2  Bolus volume: Non-significant (*P* = 0.287)  Individual: *P* < 0.0001 | | Diet: Non-significant (*P* = 0.059), Effect size 𝜂^2^ = 0.01  Bolus volume: Non-significant (*P* = 0.070)  Individual(DT): *P* < 0.0001; Individual(PT): *P* < 0.0001 | |

**Table S6.** Relative duration of each phase of epiglottal inversion (descent, laryngeal vestibule closure LVC, ascent) during drinking behavior in 12- and 16-week old pigs. Table entries are group means (bold) and individual average ± standard deviation. For each variable, the effect of the transition strategy (direct versus progressive) was assessed by testing whether the between-group difference was significant. Bolus volume was included as a covariate, and between-individual differences were also tested.

| **Drinking liquid** | **12 weeks** | | **16 weeks** | |
| --- | --- | --- | --- | --- |
|  | **All individuals** | | **DT** | **PT** |
| **Epiglottal descent duration**  % total epiglottal movement duration | **28.0% ± 9.3** | | **30.8% ± 10.3** | **25.9% ± 7.2** |
|  | 43: 33.2% ± 7.2  48: 35.0% ± 8.6  53: 21.7% ± 5.2  54: 33.1% ± 10.5  55: 23.3% ± 6.0 | 40: 33.5% ± 8.2  45: 30.8% ± 10.0  49: 18.1% ± 5.8  52: 27.0% ± 5.5  56: 29.1% ± 8.2  57: 28.6% ± 6.5 | 43: 31.2% ± 6.4  48: 38.7% ± 8.9  53: 30.7% ± 11.7  54: 27.1% ± 4.9  55: 23.4% ± 6.4 | 40: 32.2% ± 5.9  45: 26.5% ± 9.1  49: 17.4% ± 5.5  52: 25.5% ± 6.4  56: 25.3% ± 6.4  57: 29.4% ± 6.1 |
|  | Diet: Non-significant (*P* = 0.701), Effect size 𝜂^2^ = 0.02  Bolus volume: *F*_1,437_ = 14.03, *P* = 0.0002  Individual: *P* < 0.0001 | | Diet: *F*_1,290_ = 23.22, *P* < 0.0001, Effect size 𝜂^2^ = 0.07  Bolus volume: *F*_1,290_ = 5.51, *P* = 0.020  Individual(DT): *P* < 0.0001; Individual(PT): *P* < 0.0001 | |
| **Laryngeal vestibule closure duration**  % total epiglottal movement duration | **56.7% ± 10.3** | | **58.2% ± 10.2** | **60.4% ± 7.8** |
|  | 43: 55.2% ± 7.2  48: 48.6% ± 9.8  53: 62.2% ± 8.3  54: 51.4% ± 10.3  55: 61.7% ± 6.8 | 40: 52.3% ± 8.8  45: 53.7% ± 10.1  49: 70.7% ± 5.7  52: 51.9% ± 6.5  56: 58.1% ± 8.9  57: 53.9% ± 6.8 | 43: 54.9% ± 10.3  48: 52.9% ± 11.9  53: 61.5% ± 8.4  54: 60.0% ± 8.4  55: 63.1% ± 7.3 | 40: 55.1% ± 5.4  45: 57.7% ± 4.2  49: 72.7% ± 4.9  52: 59.1% ± 5.4  56: 61.3% ± 5.9  57: 57.3% ± 7.0 |
|  | Diet: Non-significant (*P* = 0.829), Effect size 𝜂^2^ = 5.5e-3  Bolus volume: *F*_1,435_ = 12.45, *P* = 0.0004  Individual: *P* < 0.0001 | | Diet: *F*_1,290_ = 4.64, *P* = 0.032, Effect size 𝜂^2^ = 0.02  Bolus volume: Non-significant (P = 0.087)  Individual(DT): *P* = 0.003; Individual(PT): *P* < 0.0001 | |
| **Epiglottal ascent duration**  % total epiglottal movement duration | **15.3% ± 5.0** | | **11.0% ± 5.0** | **13.6% ± 3.4** |
|  | 43: 11.7% ± 2.2  48: 16.5% ± 5.2  53: 16.1% ± 7.6  54: 15.5% ± 3.1  55: 15.0% ± 4.1 | 40: 14.2% ± 3.4  45: 15.5% ± 3.1  49: 11.2% ± 1.7  52: 21.2% ± 4.7  56: 12.9% ± 2.8  57: 17.5% ± 2.4 | 43: 13.9% ± 9.1  48: 8.4% ± 1.6  53: 7.8% ± 1.6  54: 12.8% ± 2.7  55: 13.5% ± 2.4 | 40: 12.7% ± 2.1  45: 15.8% ± 2.4  49: 9.9% ± 2.0  52: 15.3% ± 2.8  56: 13.4% ± 4.3  57: 13.3% ± 2.9 |
|  | Diet: Non-significant (*P* = 0.809), Effect size 𝜂^2^ = 6.9e-3  Bolus volume: Non-significant (*P* = 0.859)  Individual: *P* < 0.0001 | | Diet: *F*_1,290_ = 29.18, *P* < 0.0001, Effect size 𝜂^2^ = 0.09  Bolus volume: Non-significant (P = 0.233)  Individual(DT): *P* < 0.0001; Individual(PT): *P* < 0.0001 | |
